# Waiting time to infectious disease emergence

**DOI:** 10.1098/rsif.2016.0540

**Published:** 2016-10

**Authors:** Christopher J. Dibble, Eamon B. O'Dea, Andrew W. Park, John M. Drake

**Affiliations:** 1 Odum School of Ecology, University of Georgia, 140 East Green Street, Athens, GA 30602-2202 USA; 2 Center for the Ecology of Infectious Diseases, University of Georgia, 140 East Green Street, Athens, GA 30602-2202 USA

**Keywords:** bifurcation delay, critical slowing down, early-warning systems, emerging infectious disease

## Abstract

Emerging diseases must make a transition from stuttering chains of transmission to sustained chains of transmission, but this critical transition need not coincide with the system becoming supercritical. That is, the introduction of infection to a supercritical system results in a significant fraction of the population becoming infected only with a certain probability. Understanding the waiting time to the first major outbreak of an emerging disease is then more complicated than determining when the system becomes supercritical. We treat emergence as a dynamic bifurcation, and use the concept of bifurcation delay to understand the time to emergence after a system becomes supercritical. Specifically, we consider an SIR model with a time-varying transmission term and random infections originating from outside the population. We derive an analytic density function for the delay times and find it to be, in general, in agreement with stochastic simulations. We find the key parameters to be the rate of introduction of infection and the rate of change of the basic reproductive ratio. These findings aid our understanding of real emergence events, and can be incorporated into early-warning systems aimed at forecasting disease risk.

## 1. Introduction

The emergence and re-emergence of infectious diseases is an enormous concern to global public health. Nearly 70% of emerging human diseases have zoonotic origins [1–3], including SARS [4], MERS [5], AIDS [6,7] and Ebola [8]. Similarly, the re-emergence of previously controlled or eradicated diseases, including vaccine-preventable diseases such as measles [9] and pertussis [10], continues to be a health and economic burden [11]. Owing to factors including reductions in vaccine uptake [12], pathogen evolution [13] or changes in host demography [14], emerging and re-emerging diseases make a transition from self-limiting, stuttering chains of infection (which inevitably go extinct), to sustained chains of human-to-human transmission (major outbreaks or epidemics). This critical transition becomes possible when the average number of infections caused by a single infectious individual in an entirely susceptible population (called *R*_0_) becomes greater than 1—at this point the population is often referred to as supercritical [15,16]. Forecasting this critical transition is the goal of early-warning systems [17–19] of disease emergence [12]. In addition to knowing when a population will become supercritical [12], accurate forecasting also requires estimating the time of the first major outbreak or epidemic of a newly emerging or re-emerging disease. Crucially, major outbreaks do not happen immediately after *R*_0_ = 1. Rather, they occur with some non-zero delay—for which we do not yet have an adequate theory.

**Figure 1.**
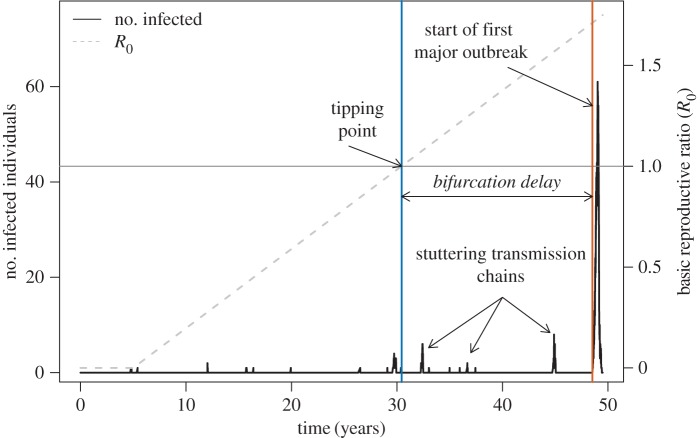
Bifurcation delay is the time between the tipping point at *R*_0_ = 1 (intersection of grey horizontal line and blue vertical line) and the beginning of an actual disease epidemic (orange vertical line, definition in text). This simulated time series is a stochastic realization of the hinge SIR model, where the transmission rate (*β*) begins to increase at a set time (here, *t* = 5 years) at rate *α*. This example contains multiple ‘sparks’ after the transition to supercriticality that do not lead to epidemics, demonstrating that bifurcation delay is not simply the waiting time for a spark to occur after *R*_0_ > 1. Other parameters for this simulation: rate of increase in transmission, *α* = 0.001 yr^−1^, initial population size (*N*_0_) = 1000, recovery rate, *γ* = 365/14 yr^−1^ (corresponding to a 14 day infectious period), spontaneous infection rate, *ξ* = 0.00067 yr^−1^, birth and death rate, *μ* = 1/60 yr^−1^. (Online version in colour.)

Waiting times between criticality and emergence imply that host populations may be at risk of a major outbreak for an extended period of time, despite experiencing only small clusters of cases (figure 1). In this way, a substantial waiting time hides the true state of a system, inhibiting our ability to intervene in response to a large, but obscured, threat. Furthermore, long waiting times in disease re-emergence could mean that reversing the driver of a critical transition to its pre-outbreak levels might not push a pathogen's *R*_0_ below 1. For instance, if reductions in vaccine uptake facilitate disease outbreaks (e.g. measles [20–22]), increasing coverage to vaccination levels that existed immediately prior to an outbreak may not reduce *R*_0_ below 1, because the transition to *R*_0_ > 1 could have occurred well before the first major outbreak. An improved understanding of the factors determining the waiting time to a major disease outbreak will facilitate epidemic forecasting.

To better relate the transition to disease emergence and the timing of major outbreaks and epidemics, we developed a model incorporating a time-varying transmission term and the acquisition of infections from outside the population. For this model, we derive an approximate density function of waiting times. For validation, this approximation is compared with stochastic simulations across a wide range of notional life-history parameters. Together, these results show that waiting times can be described by a two-dimensional distribution encapsulating the rate of infections from outside the population and the rate of change of *R*_0_. We discuss implications of our results for early-warning systems of infectious disease emergence.

## 2. Material and methods

### 2.1 Main model and concepts

Let *X* denote the number of susceptible individuals in the population; *Y*, the number of infective individuals; *Z*, the number of recovered individuals; *β*, the transmission rate; *γ*, the individual recovery rate; *μ*, the individual birth and death rate (assumed here to be equal) and *ξ*, the rate at which infections are spontaneously acquired from outside the population. The transmission rate is 0 until time *s*, at which point it increases linearly at a small rate *α*. That is, for 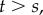 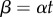. Our main model is the continuous-time Markov chain with the time-dependent rates given in table 1.

**Table 1.** Events and rate laws in our stochastic model.

event	(Δ*X*, Δ*Y*, Δ*Z*)	rate
birth	(1, 0, 0)	*μ*(*X* + *Y* + *Z*)
death of S	(−1, 0, 0)	*μX*
death of I	(0, −1, 0)	*μY*
death of R	(0, 0, −1)	*μZ*
transmission	(−1, 1, 0)	*βXY*
recovery	(0, −1, 1)	*γY*
sparking	(−1, 1, 0)	*ξX*

For our model, we consider only the case that (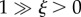). This case is of interest because in most disease emergence scenarios, there is a small influx of infectious individuals from outside the focal population—for example, due to zoonotic spillover events [23] or periodic importations of a disease from an endemic location [21]. Because of this influx, the expected number infected is necessarily greater than zero. However, the order of the expected number of cases changes qualitatively as the basic reproductive ratio passes through 1. The basic reproductive ratio is the expected number of new infections caused by an infected individual in an otherwise susceptible population, which for our model equals 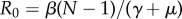, where *N* = *X* + *Y* + *Z*. For *R*_0_ < 1, the expected number infected is of order *ξN*, whereas for *R*_0_ > 1 the expected number is of order *N*. In other words, the expected number infected is approximately 1/*ξ* times larger when *R*_0_ > 1 compared with when *R*_0_ < 1. The transition from one order of magnitude to another may be referred to as an imperfect bifurcation, and the delay between this event and the time when *R*_0_ = 1 is known as a *bifurcation delay*. Our goal is to find the model parameters that largely control this delay. As we shall see, they turn out to be the rate of infection from outside of the population, which we call the *sparking rate*, and the rate of change of *R*_0_, which we call the *sweep rate*.

The results of this work are based on stochastic models for two reasons. First, an analysis of deterministic bifurcation delay in a model similar to a deterministic SIR model is already available [24]. Second, the number of infectious individuals prior to disease emergence may be small, and thus it may not be appropriate to neglect stochastic effects. We study our stochastic model and extensions of it using both simulation and analysis.

### 2.2. Simulation

Our simulation model extends our main model by including additional sources of demographic stochasticity. We make three modelling decisions to allow demographic stochasticity to have a substantial effect on delay times. First, the rate of birth in our model is always equal to the rate of death. Thus, the population size has no positive steady state, and the variance of the population size across multiple realizations of the model with the same initial population size increases over time. Second, the timing (*s*) of the increase in the transmission rate was set to year 100 to allow the variance to grow before the approach to the critical point even began. Third, the transmission rate is density dependent so that *R*_0_ varies with fluctuations in population size (table 3). Although frequency-dependent transmission is the better supported model for many diseases of interest [25,26], the direct effect of demographic stochasticity on density-dependent transmission makes it more likely to cause behaviour different from our analytic model. As the purpose of the simulation was to investigate such behaviour, and essentially ‘stress test’ the analytic results, we present results based on density-dependent transmission in the main text. A comparison of these results with those of a frequency-dependent model is included in the electronic supplementary material.

To calculate bifurcation delays in our simulation models, we first simulated trajectories of the number infected using Gillespie's direct method [27]. These simulations are not exact because the algorithm only updates the time-dependent rates at the time when state-variables are updated, but for our parameters updates should happen frequently enough that the simulations remain highly accurate. We parametrized models based roughly on the human lifespan, with a range of life-history parameters that correspond to several disease systems of emergence concern (table 2). To explore the range of possible solutions to the stochastic model, we ran 1000 stochastic simulations for each combination of parameter values. Each simulation ended at the first major outbreak, which occurred when three criteria were satisfied: (A) the size of the susceptible population was reduced below *N*/*R*_0_ (after 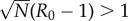, as it is difficult to distinguish epidemics from small outbreaks when *R*_0_ is only just above 1), (B) at least 15% of the population was in the recovered class, and (C) at least 2.5% of the population was infected. These criteria ensured that the disease had surpassed the long-term, deterministic endemic equilibrium number of susceptible individuals (*N*/*R*_0_) (i.e. a major outbreak occurred, A), that a substantial portion of the population had been exposed to the disease (B) and that the population was experiencing an active outbreak (C). We quantified bifurcation delay as the time between the deterministic tipping point of the system (calculated as the expected time when *R*_0_ = 1, based on the initial population size *N*_0_ and time-varying transmission rate *β*, table 3) and the last time point with 0 infectious individuals before the actual bifurcation (e.g. figure 1). To determine whether longer delays make outbreaks larger when they eventually occur, we also recorded the peak epidemic prevalence in each simulation.

**Table 2.** Parameters used for stochastic SIR simulations.

parameter	meaning	set or range of values
*μ*	host lifespan^−1^, birth rate, death rate	1/60 yr^−1^
*γ*	recovery rate	[365/5 yr^−1^, 365/41 yr^−1^]
*β*	transmission rate	if *t* < *s*, 0; otherwise *α*(*t* – *s*) individual^−1^ yr^−1^
*N*_0_	initial population size	[100 individuals, 10 000 individuals]
*ξ*	spontaneous infection rate	{1/1500 yr^−1^, 1/5000 yr^−1^, 1/10 000 yr^−1^}
*α*	rate of increase in transmission rate	[0.0005 yr^−1^, 0.01 yr^−1^]

**Table 3.** Key calculations for hinge SIR model.

value	calculation
expected time for system to become supercritical	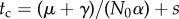
time of bifurcation	*t*_bif_ = last time before epidemic when no infectives present
bifurcation delay	*t*_bif_ − *t*_c_
basic reproductive ratio	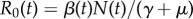
maximum infection prevalence	max(*Y*(*t*)/*N*(*t*))

### 2.3. Analysis

Our analytic model explores the effect on delay of the degree of heterogeneity in secondary cases. The degree of heterogeneity of secondary cases summarizes the consequences of heterogeneities in susceptibility, infectivity, infectious periods and contact rates. For the sake of tractability, our analytic model does not allow for demographic stochasticity in the same manner as our simulations. Our main approach is to suppose that each imported infection initiates a chain of infections which may be modelled as a branching process. The delay time is considered to be the time before the initiation of a branching process that does not go extinct. This time is a random variable that may be modelled like the time to death in a survival analysis. In the following, we derive a survival function for this time that is the basis for equations for the mean and median times given in Results.

A branching process model can be used to calculate a probability of a major outbreak that accounts for the level of heterogeneity of a given disease system. The branching process we use models the number of cases in each generation as the sum of the random number of secondary cases that are caused by each case in the previous generation. Following previous authors [28], we suppose that the secondary cases are drawn from a negative binomial distribution. The theory of branching processes then tells us that when the expected number of secondary cases is greater than 1, the probability of extinction is the smallest root of the equation2.1
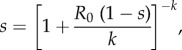
where *R*_0_ is the mean and *k* the overdispersion parameter of the negative binomial distribution. The parameter *k* allows us to tune the level of heterogeneity in the distribution of secondary cases. As *k* → ∞, the negative binomial distribution approaches a Poisson distribution, and as *k* → 0 the distribution becomes more overdispersed. For the case *k* = 1, we recover the equation that results from the assumption that cases occur at constant rate over the duration of an exponentially distributed infectious period [29]. Although equation (2.1) does not, in general, provide a closed-form equation for the extinction probability, numerically finding the smallest root of equation (2.1) is trivial. The probability of a major outbreak follows immediately as the complement of the extinction probability.

To provide a more readily understandable equation for the extinction probability, we introduce the approximation 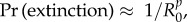 where *p* is chosen based on *k*. Electronic supplementary material, figure S1 shows the accuracy of this approximation for range of *k* and *p*. We can see that for *k* < 1, *p* ≈ *k* often has a maximum error of less than 0.1. As expected for *k* and *p* both equal to one, the calculated error is small and reflects rounding error. For *k* > 4, *p* ≈ 2 typically leads to a maximum error of less than 0.05. This approximation facilitates the following analysis.

Given a model for the parameters of the branching process, many equations describing the distribution of delay times follow readily from standard survival analysis. We first consider the model in which *R*_0_ = 1 at time zero and then increases linearly. For this model, the hazard function *λ* for a major outbreak is given by2.2
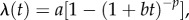
where *a* is the rate at which infections occur due to contact with other populations and *b* is the rate of change of *R*_0_ with respect to time. The parameters *a* and *b* are the sparking and sweep rates mentioned earlier. Our hazard function employs the previous approximation by letting the probability of a major outbreak, given a spark, be equal to 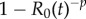. The cumulative hazard is given by2.3
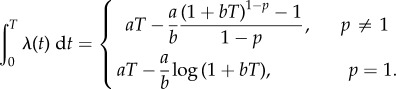


The survival function *S* computes the probability that a major outbreak has not happened before a given time, and it follows2.4
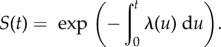
The probability density function for the delay times is the function –d*S*/d*t*. The mean delay time *E*(*T*) is given by 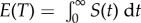. The quantile function, which provides that time at which the probability of emergence having occurred equals *F*, may be found by solving 1 − *F* = *S*(*q*) for *q*. The median delay time is equal to *q* when *F* = 1/2.

### 2.4. Comparison of analytic and simulated delay distributions

To assess the agreement between analytic and simulation results, we calculated the Kullback–Leibler (KL) divergence [30] between analytic and simulated distributions of bifurcation delay for all parameter combinations (*N* = 7200, with 1000 simulations generating each simulated distribution). KL divergence quantifies the amount of information lost (i.e. entropy) when approximating one distribution with another [30,31]. The KL divergence of the distribution with density *p* from that with density *q* is defined as 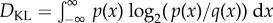. We calculated KL divergence using the entropy R package [32]. For the results presented, we used the analytic density function for *p* and the density of simulated delays for *q*. Our results are quantitatively very similar using the reverse formulation.

The KL divergence values are measured in bits. Higher values indicate more information lost via the approximation, and thus more dissimilarity between analytic and simulated distributions. In our case, it quantifies the extent to which difference in the simulation and analytic models lead to different distributions of delays.

The KL divergence of course depends on the parameters of the models. We used recursive partitioning to create a decision tree that split KL-divergence scores according to a least-squares criterion, iteratively minimizing the remaining variance below each node of the decision tree [33]. Thus, recursive partitioning allowed us to identify and visualize the parameter combinations that generated the largest discrepancies between analytic and simulated delays.

## 3. Results

For our analytic model, we obtained explicit equations for the mean and median delays for various levels of heterogeneity as well as results for the sensitivity of the delays for all levels of heterogeneity. Recall that the two parameters of our derived density function for delays are the sparking rate *a*, which is the rate at which infections are introduced from outside of the population, and the sweep rate *b*, which is the rate of change of *R*_0_. The parameter *p* indicates the degree of heterogeneity in secondary cases. For the case *p* = 1, which corresponds to the heterogeneity caused by the assumption of exponentially distributed infectious period in our simulation model, the mean follows3.1
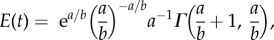
where *Γ* denotes the upper incomplete gamma function and the median is equal to3.2
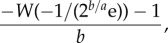
where *W* denotes the non-principal branch of the Lambert *W* function. The median is equal to3.3
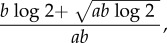
when *p* = 0.5 and3.4
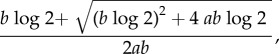
when *p* = 2. Recall that *p* = 2 corresponds to a Poisson distribution of secondary cases, whereas *p* = 0.5 corresponds to a highly overdispersed distribution of secondary cases. In the electronic supplementary material, we show that for all *p* the sensitivity of the mean and median to changes in either the sparking rate or the sweep rate depends only on the quotient of those two parameters. For relatively high sparking rates, the mean and median have a similar level of sensitivity to both parameters. As the sparking rate becomes relatively lower, the sweep rate becomes less important because for a broad range of rates the probability of an epidemic will be close to one by the time the first spark occurs. The level of heterogeneity in the distribution of secondary cases determines how quickly this transition happens by way of its effect on the relationship between *R*_0_ and the probability of an epidemic. Overall, the analytic results provide a rather simple picture of the waiting times. However, in the electronic supplementary material, we also show that the picture can be rather different if *R*_0_ changes according to a step function instead of increasing linearly. For example, the median delay has the lowest proportional sensitivity to the sparking rate when the median delay just exceeds the time of the jump in *R*_0_. In the following, we explore the extent to which the picture changes due to the elements included in our more complicated simulation models.

**Figure 2.**
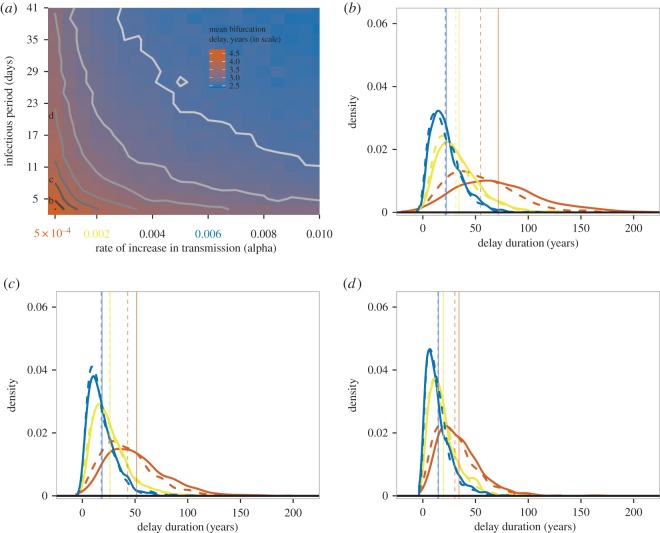
(*a*) Mean bifurcation delay (in years) decreases with increasing rate of change in the transmission rate (*α*) and increasing mean infectious period (i.e. inverse recovery rate, *γ*), as indicated by the colour of the heatmap and the contour lines (darker = more delay). Letters b–d indicate three mean different infectious periods detailed in subsequent panels. Three values of the rate of change of the transmission rate, *α*, are highlighted and explored further. (*b*–*d*) The distribution of bifurcation delay from 1000 stochastic simulations (solid lines) and expected distributions using the analytic results (dashed lines) at three rates of change in the transmission rate (*α* = 0.0005 yr^−1^, red; *α* = 0.002 yr^−1^, yellow and *α* = 0.006 yr^−1^, blue). Each panel represents a different mean infectious period (*b* = 5 days, *c* = 9 days, *d* = 21 days). Vertical lines show the mean delay for the each value of *α*, for both the observed (solid line) and expected (dashed line) delay distributions. Other life-history parameters for these examples: *N*_0_ = 1000 and *ξ* = 0.0001 yr^−1^. (Online version in colour.)

With data from stochastic simulations, we found mean bifurcation delay to decrease with increasing values of (i) sparking rate, (ii) population size, (iii) rate of increase in transmission, and (iv) infectious period (figure 2). These qualitative effects were not surprising, although the results highlight the importance of particular combinations of life-history traits. For instance, at a given value for the transmission rate's rate of change (*α*), different mean infectious periods (1/*γ*) can lead to very different predicted and observed distributions of bifurcation delay (figure 2*b*–*d*) because short infectious periods (i.e. high recovery rates) lead to lower sweep rates for a given value of *α*. The sparking rate and the sweep rate parameters of the analytic model are thus also important parameters of the simulated delay times. This parametrization also leads to a strong correlation of the delays of the frequency- and density-dependent models, although the delays of the frequency-dependent model are typically a little smaller (electronic supplementary material, figure S2). Note that the sweep rate is independent of population size in the frequency-dependent model, so the relationship between life-history parameters and delay is highly dependent on the type of transmission.

**Figure 3.**
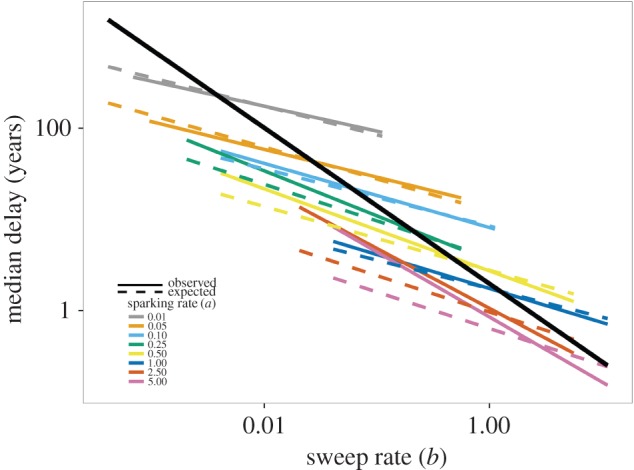
The negative relationship between bifurcation delay and the sweep rate of a system depends on the rate of sparking. The solid black line shows the overall relationship from a linear model of log(observed delay) by log(sweep rate). Solid colour lines represent the observed median bifurcation delay from model simulations, and dashed colour lines represent the expected median bifurcation delay based on analytic results. Colours show the effect of different levels of the sparking rate, *a*, on the relationship between delay and the sweep rate. Slopes and intercepts can be found in table 4. (Online version in colour.)

Closer examination of the sensitivity of the median simulated delay to changes in the sweep rate revealed both similarities and differences with our analytic model. This sensitivity may be quantified as the slope of a least-squares line in the plot of log(median delay) versus log(sweep rate). Such a slope is an estimate of the elasticity of the median with respect to the sweep rate. Consistent with the analytic results, increasing the sparking rate *a* increases the slope of the negative relationship between median simulation delays and the sweep rate (figure 3 and table 4). However, for large *a* the elasticity for the simulated delays is much more negative than −0.5, which is the minimum possible value for our analytic model (electronic supplementary material). This discrepancy is due to simulations tending to produce more delay than expected, which is in large part a consequence of the stringent conditions for an epidemic in our simulations.

**Table 4.** Scaling relationships between bifurcation delay and the sweep rate of the system. The left-most column is the linear slope of the relationship between log(delay) and log(sweep rate, *b*) for all simulations across all levels of the sparking rate *a* (e.g. solid black line in figure 3). All other columns show the slope of log(delay) by log(sweep rate) for each level of the sparking rate, *a* (e.g. colour lines in figure 3). Observed values represent results from individual stochastic simulations, and expected values for the mean and median delay arise from our analysis based on a branching process model. We fitted general linear models to each to obtain estimates of explained variance, *R*^2^.

observed delay		observed median delay			expected median delay			observed mean delay			expected mean delay		
intercept	sparking rate (*a*)	intercept	slope (log(*b*))	slope s.e.	intercept	slope (log(*b*))	slope s.e.	intercept	slope (log(*b*))	slope s.e.	intercept	slope (log(*b*))	slope s.e.
0.54	0.010	3.847	−0.289	0.008	3.743	−0.306	0.002	3.600	−0.331	0.021	4.124	−0.262	0.002
slope	0.025	3.055	−0.336	0.011	3.095	−0.310	0.003	2.893	−0.352	0.029	3.433	−0.267	0.003
−0.852	0.050	2.652	−0.311	0.009	2.528	−0.345	0.002	2.318	−0.404	0.024	2.825	−0.307	0.003
slope s.e.	0.067	2.847	−0.203	0.011	2.103	−0.408	0.003	1.790	−0.479	0.031	2.350	−0.380	0.003
0.003	0.100	2.096	−0.352	0.011	2.135	−0.313	0.003	1.953	−0.377	0.029	2.413	−0.270	0.003
	0.125	1.837	−0.405	0.011	1.764	−0.399	0.003	1.698	−0.417	0.030	2.001	−0.368	0.003
	0.167	1.684	−0.401	0.011	1.553	−0.410	0.003	1.410	−0.454	0.030	1.773	−0.383	0.003
	0.250	1.258	−0.493	0.011	1.345	−0.400	0.003	1.178	−0.489	0.030	1.559	−0.370	0.003
	0.330	1.060	−0.520	0.011	1.142	−0.412	0.003	0.944	−0.522	0.029	1.345	−0.383	0.003
	0.500	1.002	−0.450	0.009	1.020	−0.349	0.002	0.884	−0.477	0.024	1.228	−0.312	0.003
	0.670	0.596	−0.613	0.011	0.734	−0.412	0.003	0.547	−0.603	0.029	0.917	−0.383	0.003
	1.000	0.557	−0.373	0.011	0.553	−0.313	0.003	0.478	−0.384	0.029	0.733	−0.270	0.003
	2.500	0.057	−0.658	0.011	−0.037	−0.400	0.003	0.003	−0.714	0.029	0.109	−0.370	0.003
	3.330	−0.043	−0.749	0.011	−0.213	−0.412	0.003	−0.113	−0.813	0.029	−0.076	−0.383	0.003
	5.000	−0.162	−0.713	0.011	−0.453	−0.400	0.003	−0.158	−0.736	0.029	−0.327	−0.370	0.003
	6.670	−0.169	−0.824	0.011	−0.620	−0.412	0.003	−0.193	−0.814	0.030	−0.504	−0.383	0.003
*R*^2^ (adj)	0.6624		0.9921			0.9995			0.7776			0.9993	

In general, predictions based on theory derived assuming large, fixed population sizes are representative of stochastic simulations (figure 2; electronic supplementary material, figure S3). Recursive partitioning shows that approximately 78% of the variation in KL divergence can be accounted for by the sparking (*a*) and sweep (*b*) rates. Differences in predicted and observed delay distributions were greatest with slow sweep rates, fast recovery rates, high sparking rates and small population sizes (electronic supplementary material, figure S3).

**Figure 4.**
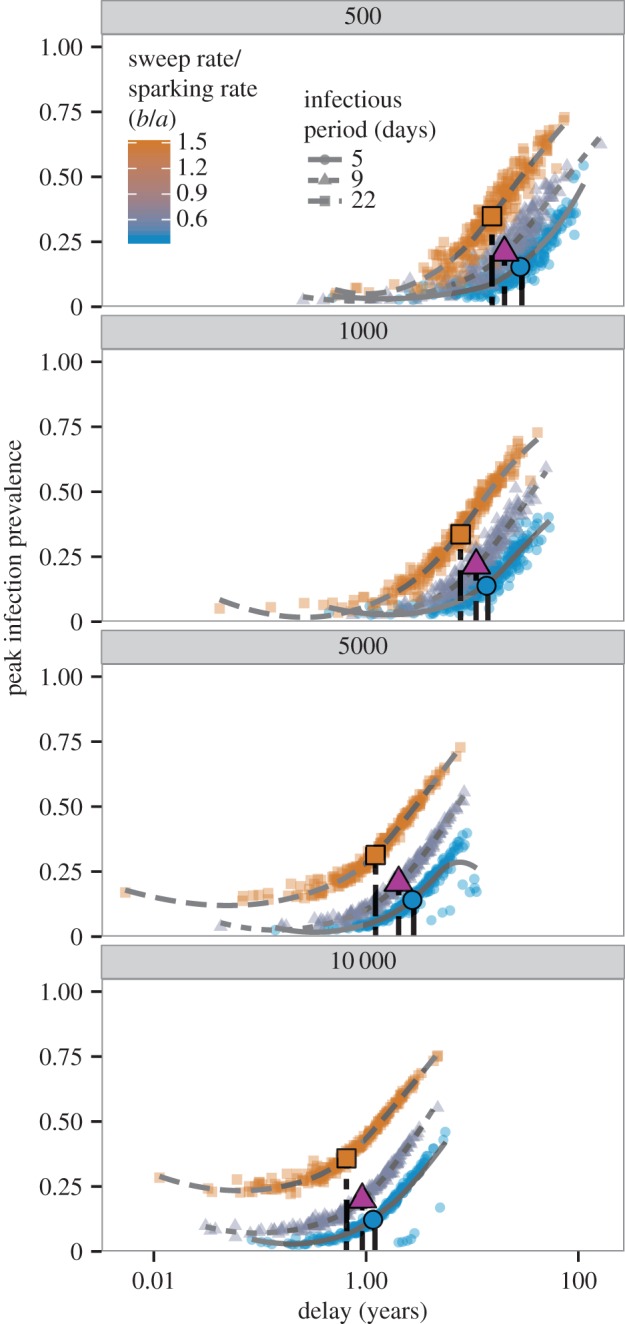
Bifurcation delay can lead to epidemics with greater peak prevalence, although this effect depends on the life-history parameters of the system. Panels show peak epidemic prevalence by stochastic realizations of bifurcation delay (*x*-axis on log-scale). Symbols and line types represent three different infectious periods, approximating three important childhood diseases (5 days for measles, 9 days for chickenpox, 22 days for pertussis). Lines are loess smooths added for visual clarity. Colour represents the ratio of *b* to *a*, which describes how quickly *R*_0_ changes relative to how frequently imported infections occur in a system. Symbols are positioned at the median of the *x*- and *y*-axes for each combination of life-history parameters. Panels show four different initial population sizes (*N*_0_ = 500, 1000, 5000 and 10 000). The relationship between delay and peak infection prevalence depends on how quickly *R*_0_ increases, which is governed by the sweep rate, mean infectious period and population size. (Online version in colour.)

For a given combination of host and pathogen life-history parameters, longer delays give rise to larger outbreaks, because *R*_0_ continues to increase ever-higher above the critical value of 1. Thus, a measles outbreak that occurs, by chance, 1 year after the population becomes supercritical will be larger, on average, than a measles outbreak that occurs six months after the population becomes supercritical (assuming the same underlying parameters). The slope of this relationship between delay and prevalence *within* a life-history parametrization depends on how quickly *R*_0_ changes relative to the sparking rate (i.e. *b*/*a*, figure 4). Note that the relationship between delay and prevalence does not necessarily hold *across* parameter combinations (i.e. when comparing different diseases; figure 4). Systems with shorter delays can have higher peak prevalence if they exhibit a longer infectious period, for instance, and thus a faster change in *R*_0_ over time (which itself contributes to differences in mean delay).

## 4. Discussion

Forecasting outbreaks of newly emerging and re-emerging diseases is difficult. Adding to this difficulty is the fact that major outbreaks of emerging diseases need not occur immediately after they become a possibility. Rather, they can exhibit bifurcation delay, the duration of which has until now remained largely undescribed. We have shown that even for models that include substantial demographic stochasticity, this delay is well described by a simple survival function with two parameters. One is the rate of transmission from outside of the population, which we call the sparking rate, and the other is the rate of change of *R*_0_, which we call the sweep rate. Further, the sensitivity of the delay to changes in these parameters depends only on their quotient. We next explain the intuition that indicates the importance of these parameters.

We found that the greater the sparking rate, the smaller the waiting time for a major disease outbreak (figure 3). This result follows Bartlett [34], who showed that very low sparking rates could generate long time lags between recurrent outbreaks. In dynamic bifurcations more generally, the addition of stochasticity or noise to slow passages through bifurcation points reduces bifurcation delay [35,36] by hastening the departure from an unstable equilibrium. While our results are in agreement, we also note that ‘noise’ in our system is strictly required for the critical transition to disease emergence. Without any infections from outside the population, a supercritical host population will never experience an outbreak or epidemic.

We also found that bifurcation delay depends on the sweep rate. This result is comparable with the phenomenon in Bartlett's model [34] where the time to a recurrent outbreak depends on both the rate of influx of susceptibles and *R*_0_. With slow sweep rates, the addition of an infectious individual to a population in the interval following the time when *R*_0_ = 1 h as a low probability of leading to a major outbreak [37]. Naturally, the expected time to the major outbreak increases in this case, and bifurcation delay in disease emergence is not simply the interval of time preceding a sparking event.

Our theoretical and simulation results captured the expected negative relationship between the duration of bifurcation delay and the sweep rate of a system [36,38]. However, we also found that the slope and intercept describing the relationship between the duration of bifurcation delay and the sweep rate depended on the sparking rate. The duration of bifurcation delay depends more on the sparking rate when the sparking rate is low (i.e. outbreaks are spark-limited), and more on the sweep rate when the sparking rate is high (i.e. driver-limited) (figure 3, see also electronic supplementary material). The degree of spark- or driver-limitation of a system reflects the size of disease outbreaks (figure 4). It may also determine the ability of early-warning systems based on critical slowing down to detect the tipping point at *R*_0_ = 1 because a driver-limited system would likely experience a series of minor outbreaks before a major outbreak. The near-critical dynamics of the minor outbreaks could trigger an early warning.

There are a number of practical consequences of bifurcation delay in disease emergence. First, delayed emergence means host populations can be supercritical for extended periods of time without experiencing a major outbreak (e.g. figure 1). Stuttering chains of transmission do not necessarily indicate, then, that *R*_0_ < 1. Without accounting for bifurcation delay, we might underestimate the likelihood of a major epidemic when transmission appears limited. This phenomenon is exhibited in many of our simulations, and is likely to occur in generic emergence time series with small clusters of cases followed by large, unexpected outbreaks. A major difficulty for disease risk assessment lies in determining whether a system with apparent subcritical transmission is truly subcritical, or actually supercritical and experiencing a delayed bifurcation.

Additionally, bifurcation delay in disease re-emergence can obscure the timing of the system becoming supercritical, and thus the level of a given driver that targeted control measures might try to achieve to ‘roll-back’ disease risk. For instance, slow reductions in vaccine uptake that contribute to a major outbreak of a vaccine-preventable disease are also subject to bifurcation delay [12]. Owing to the delay, control measures that increase vaccination coverage to what it was immediately before an outbreak may not reduce *R*_0_ below 1 because *R*_0_ may have exceeded 1 substantially earlier than the beginning of the outbreak. Further, separation in the timing of disease (re)emergence and changes in factors permitting emergence can compromise detection of statistical association between epidemic occurrence and underlying causes.

While the effects of sparking (*a*) and the probability of an epidemic given a spark (which is governed by the sweep rate, *b*) on the duration of bifurcation delay are relatively intuitive, it is worth reiterating the importance of the terms that go into each. For instance, the absolute number of infectious imports for a given system may depend on population size (as in pre-vaccine era measles [39]), which would have to be accounted for in any estimate of the sparking rate. On the other hand, unless the populations being modelled are very small (e.g. a school or village), frequency-dependent transmission is probably a better assumption for many diseases [25,26] and so the sweep rate may be largely independent of population size. Nevertheless, the same rate of change in the transmission rate for two different diseases could still be consistent with different sweep rates and thus different expectations of delay, and even different sizes of eventual disease outbreaks. For example, differences in the infectious period of childhood diseases such as measles and pertussis could mean that a constant 1% reduction in vaccine uptake per year would result in earlier, larger pertussis outbreaks, because a longer infectious period (all else being equal) generally increases *R*_0_ (figure 4).

## 5. Conclusion

Moving forward, efforts relying on the identification of critical slowing down to forecast the emergence of infectious diseases may incorporate our findings about bifurcation delay [12]. The rate of critical slowing down and the sweep rate of a system are closely related, and indicators of critical slowing down may be informative of the sweep rate. With this information and an estimate of the sparking rate from observed case data, future early-warning systems may be able to not only generate forecasts of when *R*_0_ exceeds 1, but also produce distributions of likely waiting times to major outbreaks or epidemics (e.g. figure 2*b*–*d*). This approach could also be used in hindsight, in order to better assess the timing of when *R*_0_ exceeded 1 to set the stage for previous epidemics. Our goal is that this combined framework will produce model-independent forecasts that provide actionable information about the state of an emerging disease, particularly during the transition from subcritical dynamics to sustained transmission among hosts.

## Supplementary Material

Supplemental analyses: model sensitivity, frequency-dependent transmission, and KL divergence partitioning

## Data accessibility

Simulation data have been archived at the Dryad Digital Repository doi:10.5061/dryad.dp4kb [40].
