# Supplementary material for: Waiting time to infectious disease emergence
Source: J R Soc Interface. 2016 Oct;13(123):20160540. doi: 10.1098/rsif.2016.0540 (PMC5095216; doi:10.1098/rsif.2016.0540)
Supplement: Supplemental analyses: model sensitivity, frequency-dependent transmission, and KL divergence partitioning [file rsif20160540supp1.pdf]

Supplementary material:
Waiting time to infectious disease emergence

Christopher J. Dibble   Eamon B. O'Dea   Andrew W. Park   John M. Drake

September 19, 2016

Sensitivity of the analytic model

This section further develops the analytic results regarding the distribution of the delay between R_0 exceeding 1 and a disease emerging that was introduced in the main text. As described in the main text, our analytic approach is to suppose that each imported infection initiates a chain of infections which may be modelled as a branching process. The delay time is considered to be the time before the initiation of a branching process that does not go extinct. This time is a random variable that may be modelled like the time to death in a survival analysis. In the main text, we derived a survival function for this time and equations for the mean and median delay time for particular levels of heterogeneity in the distribution of secondary cases. The approximation with the error plotted in figure S1 was instrumental in those calculations. Here, we study the sensitivity of the mean and median survival time to changes in model parameters. We use the same notation as in the main text.

Although we do not have an explicit equation for the median for all levels of heterogeneity, we can still obtain a detailed understanding of how it responds to changes in model parameters. That is, we can describe in some detail the behaviour of the elasticities of the quantiles. The elasticity $e_{y,x}$ of a function's output y with respect to a parameter x is defined as $\frac{\partial y}{\partial x}\frac{x}{y}$. In words, it is the relative change in the function's value with respect to a relative change in one of its parameters. To derive $e_{q,a}$ we first note from the definition of S and λ that

$$a = -\log(1-F)/\left[\left(\int_0^q \lambda(u)\,\mathrm{d}u\right)/a\right], \tag{S1}$$

$$\frac{\partial a}{\partial q} = \log(1-F)(\lambda(q)/a)/\left[\left(\int_0^q \lambda(u)\,\mathrm{d}u\right)/a\right]^2, \tag{S2}$$

$$\frac{\partial a}{\partial q} = \lambda(q)a/\log(1-F), \tag{S3}$$

$$\frac{\partial q}{\partial a} = \log(1-F)/[\lambda(q)a]. \tag{S4}$$

Thus, $e_{q,a} = \log(1-F)/[\lambda(q)q]$. It is instructive to view that equation as, in light of the definitions given the main text, stating that $e_{q,a}$ is equal to minus the area under the hazard curve from 0 to q divided by the area of a rectangle with sides of length $\lambda(q)$ and q. As a grows large, q grows small and the hazard function from 0 to q approaches a straight line. Therefore, as $a \to \infty$, $e_{q,a} \to -1/2$, the area of a triangle divided by that of a rectangle. As a decreases, q grows large. The ratio of $\lambda(q)$ to $\lambda(q/x)$ for any positive x decreases. Stated differently, $\frac{\mathrm{d}}{\mathrm{d}t}\frac{\lambda(t)}{\lambda(tx)} > 0$. To prove this fact, note that the following statements are logically equivalent:

$$\frac{\lambda(tx)}{\lambda(t)} > \frac{\lambda'(tx)}{\lambda'(t)}, \tag{S5}$$

$$[(1+btx)^p - 1](1+btx) > x[(1+bt)^p - 1](1+bt). \tag{S6}$$

The function $[(1+bt)^p - 1](1+bt)$ is convex and passes through the origin. Therefore, the line passing through the origin and the function's value at t lies below the function at tx. That proves inequality (S6).

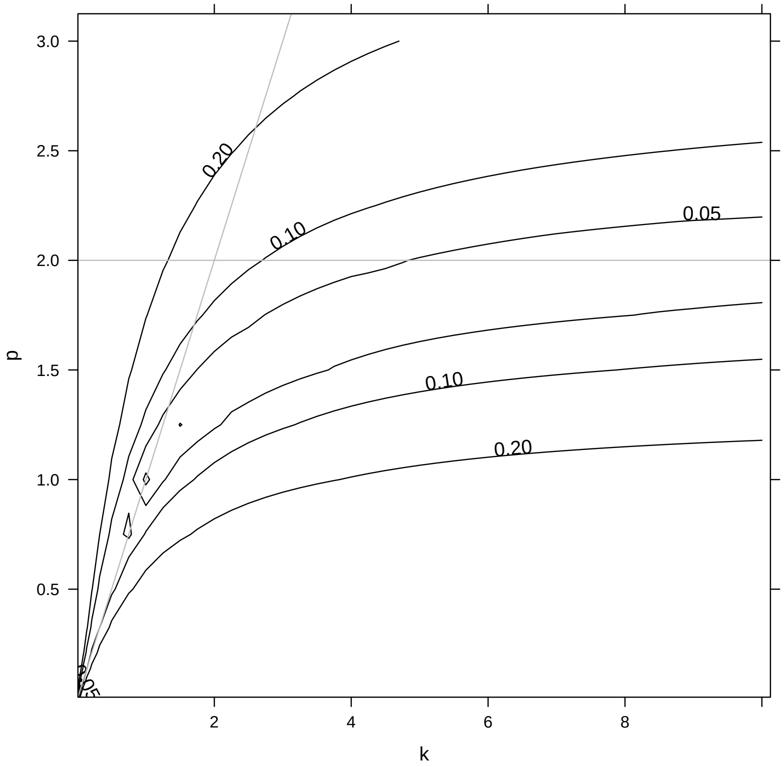

Figure S1: Maximum difference between the exact probability of extinction based on the fixed point of $s = [1 + R_0(1 - s)/k]^{-k}$ and the approximation R_0^{-p} over the range of R_0 from 0 to 200. The grey lines are drawn to point out that $p = k$ results in good approximations for low k and $p = 2$ results in good approximations for high k.

Consequently, for all p the area under the hazard curve approaches $q\lambda(q)$ as q increases. It follows that $e_{q,a}$ is an increasing function of a that asymptotically approaches -1 as $a \to 0$. The parameter p controls the rate at which this happens by determining how quickly λ approaches its limit. In summary, the quantiles become more sensitive to the sparking rate as it decreases, and this happens more quickly when the level of overdispersion is low.

The elasticity $e_{q,b}$ of the quantiles with respect to the rate of increase of R_0 is closely related to $e_{q,a}$. To see this, observe that the quantiles q are homogeneous functions of degree -1 of both a and b. That means that if a and b are both multiplied by a factor x then any quantile is multiplied by x^{-1}. This statement can be verified by observing that jointly adjusting a, b and q in that manner does not change the value of the survival probability. Euler's homogeneous function theorem [1] then tells us that

$$-q(a,b) = \frac{\partial q}{\partial a}a + \frac{\partial q}{\partial b}b. \tag{S7}$$

Consequently, from the definition of the elasticities $e_{q,b} = -1 - e_{q,a}$. Based on our knowledge of $e_{q,b}$, we can then say that the $\lim_{a\to\infty} = -1/2$ and that as a decreases, $e_{q,b}$ increases asymptotically toward 0.

So far, we have only made statements about how the elasticities change with a, but it turns out that the elasticities depend only on the quotient a/b. In other words, they are homogeneous functions of degree 0 of a and b. This fact can be verified by determining from equation (S7) that the partial derivatives in the elasticities are homogeneous functions of degree -2. Thus our previous statements about how the elasticities change as a increases (while b is held constant) can be converted directly into statements about how the elasticities change as b decreases (while a is held constant).

The elasticities of the mean with respect to a behaves similarly to those of the quantiles. To show this, we first note that

$$\frac{\partial\,\mathrm{E}(T)}{\partial a} = \frac{\partial}{\partial a}\int_0^\infty S(t)\,\mathrm{d}t \tag{S8}$$

$$= -\int_0^\infty \int_0^t \lambda(u)\,\mathrm{d}u\,S(t)\,\mathrm{d}t/a. \tag{S9}$$

Then

$$e_{\mathrm{E}(T),a} = \frac{-\int_0^\infty \int_0^t \lambda(u)\,\mathrm{d}u\,S(t)\,\mathrm{d}t}{\mathrm{E}(T)} \tag{S10}$$

$$= \frac{-\int_0^\infty \int_0^t \lambda(u)\,\mathrm{d}u\,S(t)\,\mathrm{d}t}{\int_0^\infty t\lambda(t)S(t)\,\mathrm{d}t}. \tag{S11}$$

As with $e_{q,a}$, a geometric interpretation is instructive. $e_{\mathrm{E}(T),a}$ is the result of dividing one weighted average of areas by another. The weights are $S(t)$ and are shared by the dividend and divisor. As with $e_{q,a}$, the area in the dividend is the area under the hazard curve and the area in the divisor is that of a rectangle. As a grows large, S declines rapidly with t and thus only the areas with t near 0 make a substantial contribution to the averages. Since λ from 0 to t approaches a straight line as $t \to 0$, $e_{\mathrm{E}(T),a}$ approaches the average area of triangles divided by the average areas of corresponding rectangles, which is 1/2. As t increases, as explained previously the areas under the hazard function becomes more rectangular with a rate of convergence determined by p. Thus $e_{\mathrm{E}(T),a}$ is an increasing function of a and asymptotically approaches -1 as $a \to 0$. Recall that we found the same limits and monotonicity for $e_{q,a}$.

We can in fact go on to show that all of the limits and monotonicity results found for elasticities of the quantiles apply to elasticities of the mean. Recall that these results were consequences of the limits and monotonicity of $e_{q,a}$ as a function of a and that $q(a,b)$ was a homogeneous function of degree -1. To see that $\mathrm{E}(T)$ as a function of a and b is homogeneous of degree -1, consider that $\int_0^\infty S(t, ax, bx)\,\mathrm{d}t = \int_0^\infty S(t/x, ax, bx)\,\mathrm{d}t/x = \int_0^\infty S(t, a, b)\,\mathrm{d}t/x = \mathrm{E}(T)/x$. The limits and monotonicity results for the quantiles thus carry over to the mean. These results are contingent on modelling R_0 as a linear function, however.

If R_0 is modelled with a step function, the elasticities can be quite different. For example, let

$$\lambda(t) = \begin{cases} a(1 - R_{0,1}^{-p}), & t < t_1 \\ a(1 - R_{0,2}^{-p}), & t_1 \le t, \end{cases} \tag{S12}$$

where $1 < R_{0,1} < R_{0,2}$. The quantile function is given by

$$q(a,t_1) = \begin{cases} \frac{-\log(1-F)}{a(1-R_{0,1}^{-p})}, & -\frac{\log(1-F)}{a(1-R_{0,1}^{-p})} < t_1 \\ \frac{-\log(1-F)-(at_1R_{0,2}^{-p}-at_1R_{0,1}^{-p})}{a(1-R_{0,2}^{-p})}, & \text{otherwise.} \end{cases} \tag{S13}$$

The elasticity of the quantiles with respect to a are then

$$e_{q,a} \begin{cases} -1, & -\frac{\log(1-F)}{a(1-R_{0,1}^{-p})} < t_1 \\ \frac{R_{0,2}^{-p}-R_{0,1}^{-p}}{-\log(1-F)/(at_1)-(R_{0,2}^{-p}-R_{0,1}^{-p})} - 1, & \text{otherwise.} \end{cases} \tag{S14}$$

We can see from these equations that $e_{q,a}$ starts at -1 and stays there until the median is equal to t_1. Then $e_{q,a}$ jumps away from -1 toward zero and relaxes back toward -1 as a further decreases. Note that the $q(a,t_1^{-1})$ is a homogeneous function of degree -1, so $e_{q,t_1^{-1}} = -1 - e_{q,a}$. So the comparable transformation to multiplying b in the linear model by x is to divide t_1 in the step model by x. If that is done and a is multiplied by x, in both cases the quantiles will be divided by x. In summary, knowledge of the correct model for R_0 can be essential for predicting the effect of changes in parameters on delay times. In the main text, we explore the effects of other model violations such as finite population size.

Frequency-dependent transmission

The simulation results presented in the main text are based on the assumption that pathogen transmission increases with host population size (i.e. density-dependent transmission, as we also implicitly assume identical areas). With frequency-dependent transmission, typical of vector-borne diseases, STDs, and also some childhood illnesses, transmission rates are largely independent of host population density [2]. To compare bifurcation delay in simulations with each type of transmission, we created a frequency-dependent transmission model by having the rate of transmission events be equal to $\beta XY/N$ instead of βXY and keeping the equations for all of the other rates identical to those of table 1 in the main text.

The frequency-dependent model has a different equation for R_0, which must be considered when calculating the sweep rate. It has $R_0 = \beta/(\gamma + \mu)$ and thus the sweep rate is $b = \alpha/(\gamma + \mu)$. Note that for the same α, γ, and μ; the sweep rate in the density-dependent model would be a factor of N larger, which could have a profound effect on the length of the delay.

We compared the transmission models by plotting the mean (+/- SE) of simulated bifurcation delay in each against each other (500 simulations for frequency-dependent, 1000 simulations for density-dependent transmission), conditioned on the range of life history parameters presented in table 2 of the main text. For the frequency-dependent model, the rate of change of the transmission rate, α, was set to be a factor of N_0 larger to make the sweep rate comparable to that of the density-dependent model with the same life-history parameters. Figure S2 shows that mean delay is generally similar over the range of sparking and sweep rates. However, it is apparent that for the shorter delays, the frequency-dependent transmission model produces, on average, less bifurcation delay than the density-dependent model. The degree of departure depends on the sparking (a) and sweep (b) rates of the system.

Recursive partitioning of KL-divergence

We used recursive partitioning to identify the life history parameters that contributed to the largest departures of our observed delay values from our expected delay values (i.e. the Kullback-Leibler divergence [3]) (rpart package in R; [4]). We created two partitioning schemes: the first used the life history parameters initial population size (N_0), infectious interval (reciprocal of the recovery rate, γ), linear rate of increase in the transmission rate (α), and the spontaneous rate of infection from outside the population (ξ). The second used the expected sparking rate, $a = \xi N_0$, and the expected sweep rate, $b = \alpha N_0/(\gamma + \mu)$, where μ is the host birth and death rate. Each partition tree was pruned by removing uninformative partitions, as identified by cross validation plots [4]. We present here the pruned partitioning trees for each approach, as well as an estimate of the explained variance.

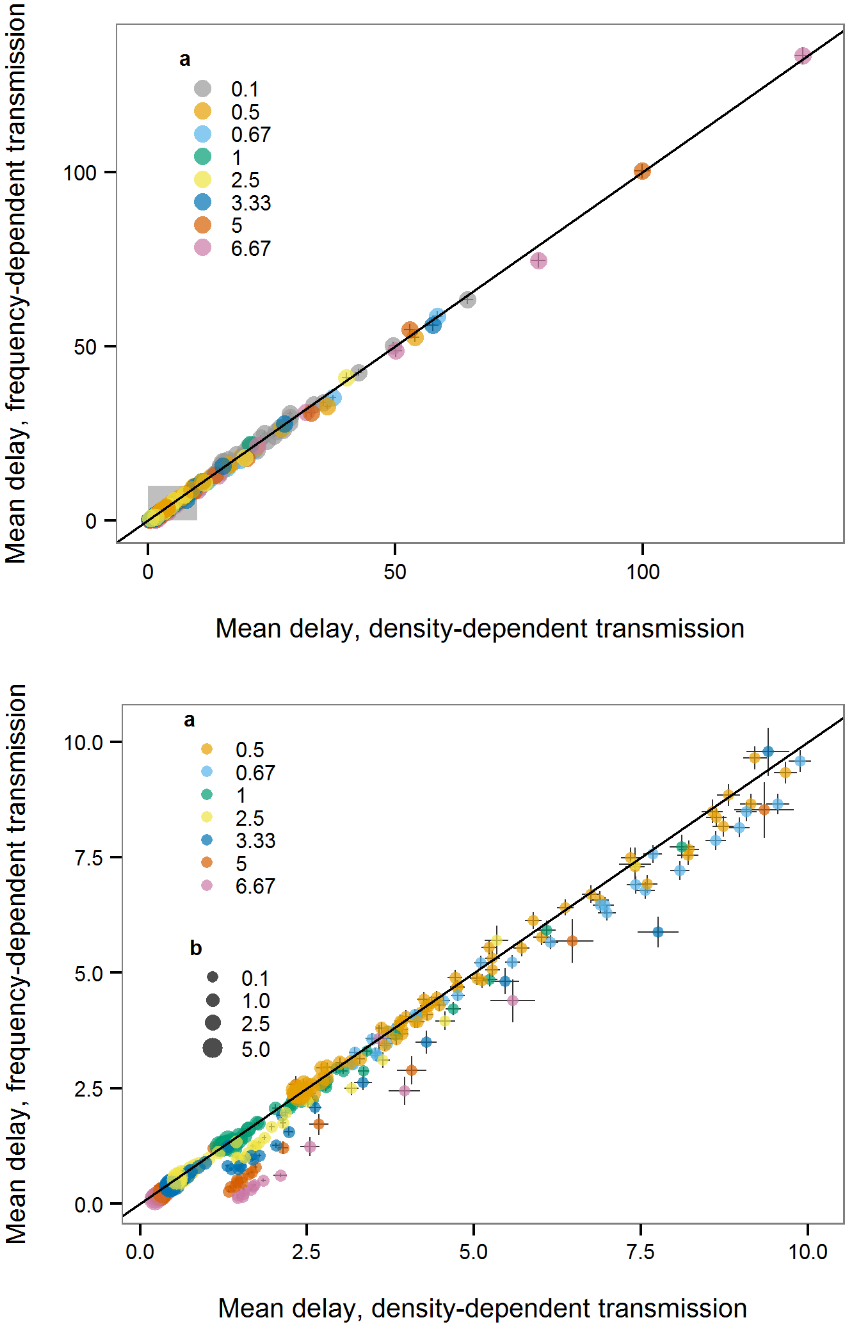

Figure S2: Mean bifurcation delay (in years, +/- SE) in stochastic simulations assuming frequency-dependent disease transmission (y-axis) is similar to mean bifurcation delay in density-dependent transmission models (x-axis). Each point represents a unique, matched combination of life history parameters; thus, points on the solid, diagonal line 1:1 line indicate identical mean delay in simulations assuming each transmission model. Symbol colours represent levels of the sparking rate, a. The mean delay was < 10 years for most of the parameters tested, which fall into the gray shaded rectangle. The lower panel is the same data but with axes adjusted to zoom in on the shaded rectangle in the upper panel. Departures from the 1:1 line show that mean delay is generally lower with frequency-dependent than with density-dependent transmission.

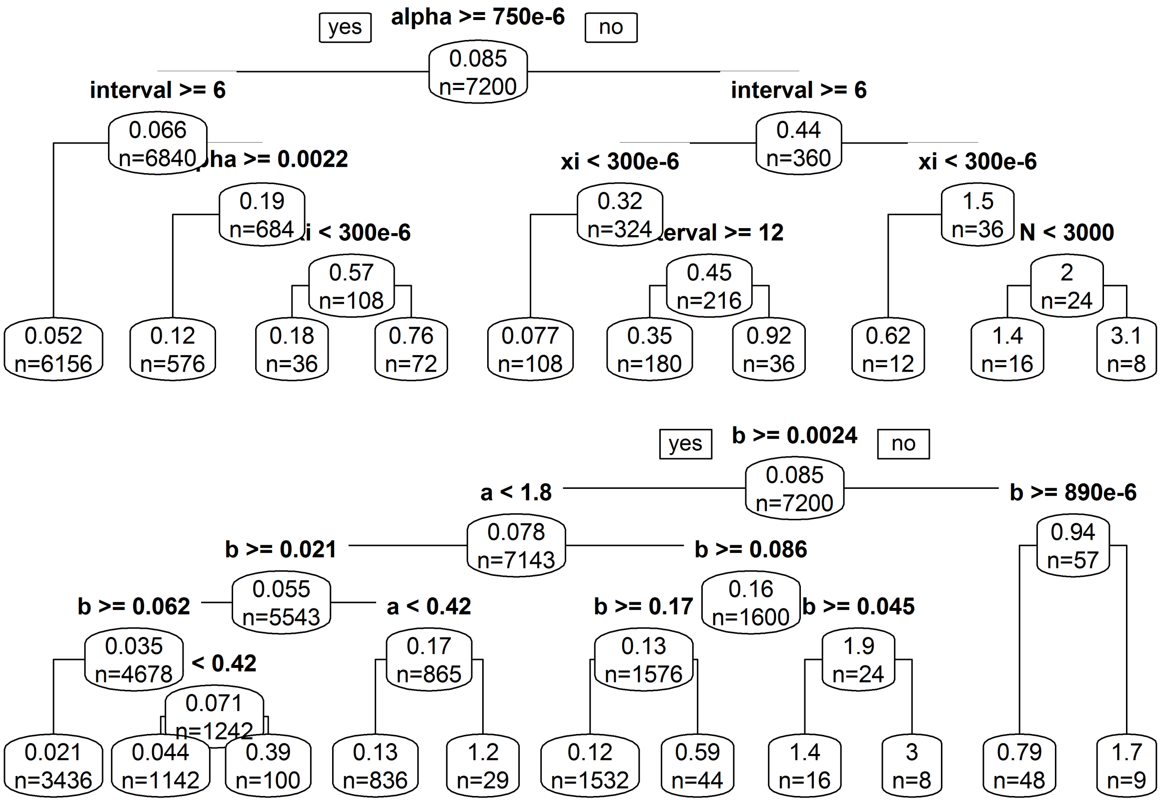

Figure S3: Recursive partitioning of the Kullback-Leibler (KL) divergence, representing the amount of information lost (in bits) when approximating the analytic distribution of bifurcation delay with that of the stochastic simulations. Each node of the partition tree has a label identifying the split, or inequality, implemented at that level, the sample size of observations, and the mean KL divergence at that level; branches extending to the left of each node satisfy the inequality (i.e. “yes”), those to the right do not (“no”). Thus, the first node in panel **a** gives the mean KL divergence (0.085 bits) of all 7200 simulations. Further nodes and branches detail the life history parameter combinations that give rise to the greatest KL divergence. “interval” refers to the infectious period of the simulated pathogen (in days), and is the reciprocal of the recovery rate, gamma. “xi” is the spontaneous rate of infection from outside the population, and “N” is the initial population size. Panel **a** shows the recursive partitioning tree based on life history parameters N, gamma, alpha, and xi (denoted N_0, γ, α, and ξ in-text), which explains 66.7% of variance in KL divergence. Panel **b** shows the tree based on the sparking and sweep rates, which explains 78% of variance in KL divergence.

References

[1] Diekmann O, Heesterbeek H, Britton T, 2012 *Mathematical Tools for Understanding Infectious Disease Dynamics.* Princeton, New Jersey: Princeton UP

[2] Keeling MJ, Rohani P, 2008 *Modeling Infectious Diseases in Humans and Animals.* Princeton, New Jersey: Princeton UP

[3] Kullback S, Leibler RA, 1951 On Information and Sufficiency. *Ann. Math. Statist.* **22**, 79–86. doi: 10.1214/aoms/1177729694

[4] Therneau T, Atkinson B, Ripley B, 2014. rpart: Recursive Partitioning and Regression Trees. R package version 4.1-8.
